# Personal

**Published:** 1907-02

**Authors:** 


### PERSONAL.

Dr. Alvah H. Doty has been reappointed Health Officer of the Port of New York. Governor Hughes makes his renomination in view of Dr. Doty's valuable public service to the state during the twelve years in which he has held the office.

A statement from the executive chamber said of Dr. Doty's service:

Dr. Doty was first appointed by Governor Morton in 1894, being then chief inspector of the division of contagious diseases in the Department of Health. He has served continuously as Health Officer ever since. Previous to his appointment the quarantine regulations had been managed in such a way that New York City was often threatened with contagious diseases brought in through the port. Dr. Doty's knowledge of the management of infectious and quarantinable diseases has prevented the recurrence of such dangers. By virtue of his office the Health Officer is a member of the Board of Health of New York City, and in this capacity Dr. Doty has rendered valuable services.

---

Dr. John Fitzgerald Fairbairn, of Buffalo, has returned from Vienna where he has been engaged in special research work for the past 18 months. He served as volunteer assistant under Hofrat Politzer and Docent Koschin, of the University of Vienna, and also visited the larger ear, nose and throat clinics of Europe. He has opened offices at 155 Allen street (corner of Park street) and will limit his practice to diseases of the ear, nose and throat. Houre: 9 to 1 and 5 to 6; Sundays by appointment. Telephones: Bell, Tupper 1076; Frontier, 10261.

---

Dr. W. W. Keen, for nearly half a century closely identified with medical institutions of Philadelphia, resigned on January 3, 1907, as professor of surgery in the Jefferson Medical College with which he had been associated for 27 years. Upon the acceptance of the resignation the trustees of the institution elected

Dr. Keen emeritus professor of surgery. Dr. Keen's resignation is to take effect on March 15, after which he will sail for a year's tour abroad.

---

Dr. L. T. Hess, captain surgeon, United States Army, has arrived at Fort Porter to take station as post surgeon. Dr. Hess came from Fort Lawton, Washington, relieving Dr. N. W. Wilson, of Buffalo, who has been acting as post surgeon since the departure of Dr. P. C. Fauntleroy, captain surgeon, United States Army, who was ordered to Cuba with the army of occupation.

---

Dr. Ettore Marchiafava, who succeeded the late Dr. Lapponi as private physician to the Pope, was born fifty-two years ago at Civita Vecchia, and was principally educated in Rome, where he is now professor of pathological anatomy at the university. He is the discoverer of the malarial parasite.

---

Dr. John Young Brown, superintendent and surgeon in charge of the St. Louis City Hospital, read a paper on "Some further observations on the surgery of the diaphragm" before the medical society of Wayne county, Michigan, at Detroit, on January 28, 1907.

---

Dr. Archibald D. Carpenter, 69 E. Parade Avenue, Buffalo, has been appointed civil service commissioner, vice Dr. Stephen Y. Howell, resigned.

---

Dr. S Goldberg, of Buffalo, was elected president of the Cosmopolitan Club, January 10, 1907. Dr. George Roberts was elected treasurer.

---

Dr. Charles A. Clements, of Buffalo, has resigned from the board of directors of the City Hospital for Women.

---

Dr. Charles G. Stockton, of Buffalo, sailed January 19, 1907, for a tour in Europe.

